# The emerging roles of long non-coding RNA in host immune response and intracellular bacterial infections

**DOI:** 10.3389/fcimb.2023.1160198

**Published:** 2023-04-21

**Authors:** Aryashree Arunima, Erin J. van Schaik, James E. Samuel

**Affiliations:** Department of Microbial Pathogenesis and Immunology, School of Medicine, Texas A&M University, Bryan, TX, United States

**Keywords:** long non-coding RNAs (lncRNAs), host-pathogen interaction, obligate intracellular pathogens, microbiota, inflammation, phagocytes, biomarker

## Abstract

The long non-coding RNAs (lncRNAs) are evolutionarily conserved classes of non-coding regulatory transcripts of > 200 nucleotides in length. They modulate several transcriptional and post-transcriptional events in the organism. Depending on their cellular localization and interactions, they regulate chromatin function and assembly; and alter the stability and translation of cytoplasmic mRNAs. Although their proposed range of functionality remains controversial, there is increasing research evidence that lncRNAs play a regulatory role in the activation, differentiation and development of immune signaling cascades; microbiome development; and in diseases such as neuronal and cardiovascular disorders; cancer; and pathogenic infections. This review discusses the functional roles of different lncRNAs in regulation of host immune responses, signaling pathways during host-microbe interaction and infection caused by obligate intracellular bacterial pathogens. The study of lncRNAs is assuming significance as it could be exploited for development of alternative therapeutic strategies for the treatment of severe and chronic pathogenic infections caused by *Mycobacterium*, *Chlamydia* and *Rickettsia* infections, as well as commensal colonization. Finally, this review summarizes the translational potential of lncRNA research in development of diagnostic and prognostic tools for human diseases.

## 1 Introduction

The genomes of simplest micro-organisms to complex genome of humans encode a vast repertoire of biological information that needs to be unraveled at the molecular level. Next-generation sequencing and recent developments in computational programming and annotation methods have expanded our understanding of DNA beyond the protein-coding regions of the genome (Liu et al., 2013; Uszczynska-Ratajczak et al., 2018). A comparison between the genomes of different species showed functional, evolutionary, and genomic similarities in their protein-coding regions. This prompted a paradigm shift from mRNA-centric view of host gene regulation to the non-coding regions of the genome. Recent studies and consortiums like ENCODE (The Encyclopedia of DNA Elements) and FANTOM (The Functional Annotation of the Mammalian Genome) have characterized a section of the non-coding regions of the genome and identifies their regulatory role in host transcriptome (Chen et al., 2017). Among such loci are the long non-coding RNAs (lncRNAs) (Uszczynska-Ratajczak et al., 2018).

LncRNAs are classically defined as transcripts of at least > 200 nucleotides in length (Uszczynska-Ratajczak et al., 2018). The recent GENCODE v. 42 release shows out of 252,416 annotated transcripts, 89,305 are mRNA, while 57,936 are annotated lncRNAs in the human genome (Frankish et al., 2021). New tools like single cell sequencing, rapid amplification of cDNA ends sequencing (RACE-seq), capture long sequencing (CLS) method, photoactivatable ribonucleotide-enhanced cross-linking and immunoprecipitation (PAR-CLIP), capture hybridization analysis of RNA targets (CHART) and *in vitro* RNA antisense purification (RAP) have expanded our understanding of lncRNA transcriptome and interactome (Agliano et al., 2019). The tools have validated these transcripts and annotated their genomic location and structure (Chen et al., 2017). Studies have shown lncRNAs modulate diverse biological pathways at transcriptional, post-transcriptional and epigenetic levels by acting as decoys, scaffold, guides, sponges or enhancers (Wang and Chang, 2011; St.Laurent et al., 2015). As a result, they play a critical role in diverse cellular processes like embryonic development, stem cell pluripotency, neuronal and cardiac differentiation (DiStefano, 2018). They have also been linked to several cancer, immune response regulation, host-pathogen interaction and microbiota interaction (DiStefano, 2018; Agliano et al., 2019).

The current review has two main objectives. The first objective is to understand the organization of lncRNAs in human genome and how they are functionally classified and regulated. Often, the lncRNAs are spatio-temporally regulated and their genome location is biologically linked with the cellular pathways they are likely to influence in the host (Herriges et al., 2014; Dempsey et al., 2018). The second objective is to shed light on the role of several annotated and discovered lncRNAs that regulate the immune responses and pathogenesis during host-pathogen interaction with a special focus on the obligate intracellular pathogens. The review also identifies gap in lncRNA research and discusses lncRNA based therapeutic approaches in diagnosis and treatment of infectious diseases.

## 2 Classification of LncRNA

The classification of lncRNAs is mostly empirical and so far, there are no standard parameters to classify them. However, lncRNAs can be classified according to different features as follows: (A) genome location; (B) mode of action on DNA sequence. The most widely used classification of lncRNAs is based on their positioning in the genome which could be sub-divided into five classes of lncRNA (Ma et al., 2013; St.Laurent et al., 2015).

### 2.1 Based on genome location

#### 2.1.1 Intergenic and intronic lncRNAs

**Figure 1:**
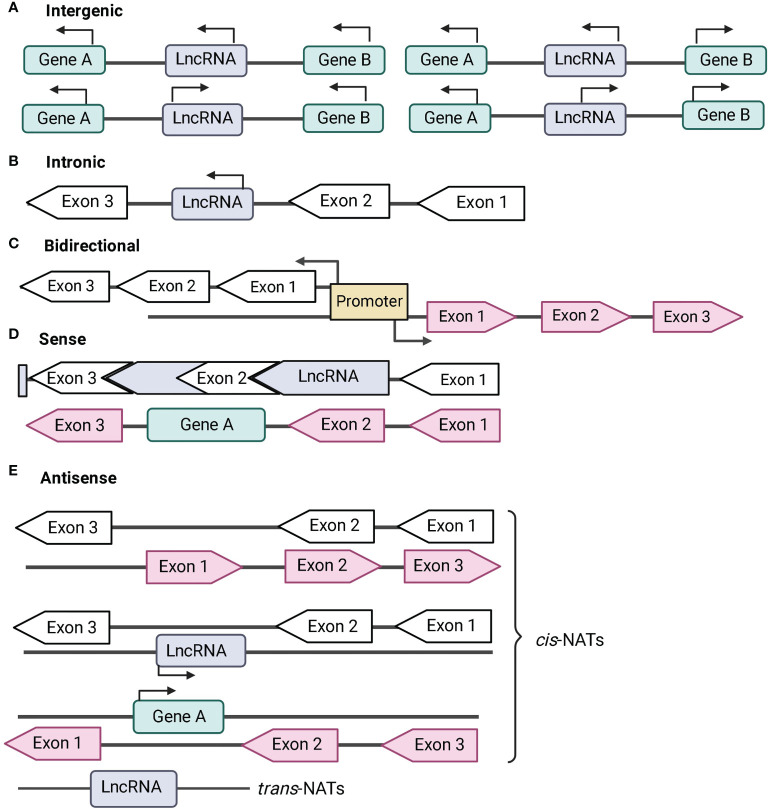
Classification of long non-coding RNA (lncRNA) based on genomic location. Protein-coding genes are represented in green and their exons are represented in black-bordered, arrow-shaped box. Introns are denoted by solid black lines. LncRNAs are represented in blue and their exons are represented in pink-arrow-shaped box. Direction of transcription is noted by the direction of the arrows. **(A)** Intergenic lncRNAs are transcribed from the intergenic regions of both the strands. **(B)** Intronic lncRNAs are transcribed from introns of protein-coding genes. **(C)** Bidirectional lncRNAs are located head-to-head with a protein-coding gene within 1 kb under a common bidirectional promoter. **(D)** Sense lncRNAs are transcribed from the sense strand of the protein-coding genes and contain exons from protein-coding genes. The lncRNA may partially overlap with the protein coding genes or may entirely overlap with the protein-coding gene through introns. **(E)** Antisense lncRNAs or naturally antisense transcripts (NATs) are transcribed from the antisense strand of protein-coding genes. NATs comprises of *cis*-NATs and *trans*-NATs. *cis*-NATs can overlap with exons or introns or may entirely overlap with the protein-coding sequence. *trans*-NATs is complementary to a target protein-coding gene and it is located in distal part of the genome.

LncRNAs that are located and transcribed from different intergenic regions are designated intergenic lncRNAs (**Figure 1A**). LncRNAs transcribed from introns of protein-coding genes are designated intronic lncRNAs (**Figure 1B**). Long-intergenic non-coding RNAs (lincRNAs) are the most widely studied type of lncRNAs. They are transcriptionally activated in a mechanism similar to mRNA and are more conserved across species than intronic lncRNA (Khalil et al., 2009; Guttman et al., 2010; Ma et al., 2013) and antisense transcripts (Guttman et al., 2010; Ma et al., 2013). Their expression is more tissue-specific and stable as compared to other types of lncRNAs (Guttman et al., 2010; Cabili et al., 2011).

#### 2.1.2 Bidirectional lncRNAs

Bidirectional lncRNAs are located head-to-head within 1kb of protein-coding genes, are transcribed under a bidirectional promoter (**Figure 1C**). This results in similar expression pattern between the lncRNA transcripts and its protein-coding gene (Carpenter et al., 2013; Atianand et al., 2017). LincRNAs and bidirectional lncRNAs do not overlap with other genes, and are therefore called as non-overlapping lncRNAs (Statello et al., 2021). Bi-directional or divergent promoters of lncRNAs are predicted as sites for evolutionary divergence to new genes or for *cis*-regulation of protein-coding genes.

#### 2.1.3 Sense lncRNAs

Sense lncRNAs are a type of overlapping lncRNAs that are transcribed from the sense strand of protein-coding genes containing exons (**Figure 1D**). Interestingly, some sense lncRNAs can function as both RNA and protein-coding gene. For instance, steroid receptor RNA activator (SRA) gene can translate into protein, and the transcript can also function as scaffold for co-activators and repressors to form complexes regulating transcription (Hubé et al., 2011).

#### 2.1.4 Antisense lncRNAs or Natural antisense lncRNAs (NATs)

Antisense or NAT lncRNAs are transcribed from the antisense strand of protein-coding genes. They are the most abundant type of lncRNAs found in the mouse and human genome (Agliano et al., 2019). NATs are further classified into *cis*-NATs and *trans*-NATs (**Figure 1E**). *cis*-NATs are complementary to its overlapping protein-coding gene, while *trans*-NATs are located in different positions of the genome and are complementary to a protein-coding gene (Ohhata et al., 2022).

### 2.2 Based on mode of action on DNA sequences

#### 2.2.1 cis-lncRNA

*cis*-lncRNAs are lncRNAs that can regulate expression of genes located in the neighboring region (Ohhata et al., 2022). Most of the lncRNA from the global transcriptome exert *cis*-regulatory effects on neighboring protein-coding gene (Engreitz et al., 2016). For instance, AS-IL1α (AS- interleukin-1 α-subunit) (Chan et al., 2015), lnc-IL7R (lnc-interleukin-7 receptor α-subunit) (Hadjicharalambous et al., 2018), and IL-1β-enhancer RNA are examples of *cis*-acting lncRNA (Engreitz et al., 2016; Robinson et al., 2020). LincRNA-Cox2 act as an enhancer RNA (eRNA) in *cis* to regulate neighboring gene *Ptgs2* (Elling et al., 2018). *Cis-*lncRNAs because of their co-occurrence with neighboring gene are ideal candidates for investigation of their biological role in host. This is usually based on the hypothesis that, *cis*-lncRNAs could be regulating the same biological pathways as their neighboring protein-coding gene (Robinson et al., 2020).

*cis*-lncRNA regulates neighboring gene either at transcriptional or epigenetic level. It represses transcription either by binding to the promoter of target gene and blocking the formation of pre-initiation complex, or by interacting with the transcription factors (TFs) (St.Laurent et al., 2015). For instance, lncRNA of DHFR (di-hydrofolate reductase) can form a stable triplex structure with the promoter region (0.8-7.3Kb region upstream) of DHFR and TFIIβ to displace the pre-initiation complex for transcriptional inhibition (Martianov et al., 2007; Ma et al., 2013). SRG1 is ~0.4-1.9Kb of size transcript that spans the promoter region of SER3 (phosphoglycerate dehydrogenase gene in *Saccharomyces cervisiae*). The resultant SRG1-RNA prevents the TF binding to *SER3* promoter to inhibit transcription (Martens et al., 2004; Ma et al., 2013). Interaction of *cis*-lncRNA with chromatin modifying enzyme (CME) complex to regulate protein-coding gene expression can be negative or positive regulatory event. Xist is a 19 Kb lncRNA which interacts with polycomb repressive complex 2 (PRC2) to trimethylate H3K27 that transcriptionally represses genes encoded on X-chromosome (Froberg et al., 2013; Maclary et al., 2017). GAL10-ncRNA (Gal10p-noncoding RNA) is ~4Kb lncRNA that recruits Rpd3S HDAC (Rpd3 small histone deacetylase complex) to reduce acetylation of H3 histone and transcriptionally represses GAL1 expression (Houseley et al., 2008; Leng et al., 2021). HOTTIP (HOXA transcript at the distal tip) is ~3.8 kb lncRNA in human that recruits MLL chromatin modifying complex to maintain a domain of active chromatin over the 5’ end of HOXA (homeobox A cluster) (Wang et al., 2011; Ghafouri-Fard et al., 2020)

#### 2.2.2 trans-lncRNA

LncRNAs that can regulate expression of genes located in different region are called *trans*-lncRNAs. For instance, lincRNA-Cox2 is upregulated (Carpenter et al., 2013) and lincRNA-erythroid prosurvival (lincRNA-EPS) is downregulated (Robinson et al., 2020) in response to Toll-like receptor (TLR)-4 and TLR-2 stimulation in murine dendritic cells (DCs) and bone marrow derived macrophages (BMDM). These lncRNAs regulate large number of distally located interferon stimulated genes (ISGs) and NF-κB regulated genes (Carpenter et al., 2013). HOTAIR (HOX antisense intergenic RNA) is a lncRNA transcribed from the HOXC (homeobox C cluster) gene locus in chromosome 12. It is exported by Suz-Twelve protein to regulate the homologous target sites at HOXD (homeobox D cluster) gene located in chromosome 2 (Rinn et al., 2007).

## 3 Biogenesis and regulation of LncRNA

The biogenesis of LncRNAs is similar to that of mRNAs and regulated by several transcriptional and epigenetic regulators. The study of the differences in biogenesis, processing and turnover of lncRNAs is important as it influences their cellular fates and functions.

### 3.1 Transcriptional processing of LncRNA

LncRNAs in comparison to mRNA contain fewer but longer exons. Their cellular expression levels are low and exhibit low primary sequence conservation. They are mainly transcribed by RNA polymerase II, but also can be transcribed by other RNA polymerases. Following transcription, they undergo splicing in a mechanism similar to that required for mRNA maturation. The matured lncRNA transcripts often comprise of 7-methyl guanosine (m_7_G) cap at their 5’ ends and polyadenylation (poly-A tail) at 3’ ends.

At the chromatin level, lncRNA and mRNA show H3K4me3 (trimethylation of histone 3 at Lys_4_) at the promoters. However, lncRNA that shows chromatin enrichment of H3K27ac (acetylation of histone 3 Lys_27_) are repressed by CME complexes; Swr1, Rsc, Ino80 and Isw2 (van Attikum et al., 2007). The initiation sites of lncRNA for transcription are divergent like most of the eukaryotic promoters. Most of the *cis*-lncRNA are transcribed antisense to the protein-coding gene by RNA polymerase II. Usually, these mRNA-lncRNA pair has coordinated expression, however transcription elongation is productive only in sense direction (Core et al., 2008). Asymmetric distribution of polyA- and splicing signals also determine elongation and stability of mRNA-lncRNA pairs. For instance, polyadenylation signals in U1 snRNP are enriched in the *cis* antisense direction, while the splicing signals are enriched in the sense direction (Almada et al., 2013).

### 3.2 Post-transcriptional processing of lncRNA

Besides, 5’-capping, 3’-polyadenylation, splicing and chemical base modification, lncRNAs undergo alternative types of processing to form mature lncRNAs. For instance, RNaseP-mediated cleavage is an alternative method of canonical cleavage and polyadenylation to process 3’ ends of most lncRNAs. MALAT1 (metastasis associated lung adenocarcinoma) and NEAT1 (nuclear enriched abundant transcript 1) are highly abundant types of nuclear lncRNA, that localizes to the nuclear speckles and paraspeckles respectively (Quinn and Chang, 2016). They have a tRNA-like-cloverleaf structure in the 3’end and A/U-rich sequence which form a stable triple helix. RNaseP cleaves tRNA-like-cloverleaf structure from the immature MALAT1 and NEAT1 to produce mature transcripts. The mature MALAT1 transcript is known as MALAT1−associated small cytoplasmic RNAs (mascRNAs) which structurally mimics tRNA and is exported to the cytoplasm (Wilusz et al., 2008). mascRNA subunit is cleaved, the U–A•U RNA triple helix at the 3′ end of mature MALAT1 increases the stability of the transcript that enables it to regulate alternative splicing (Tripathi et al., 2010; Brown et al., 2012; Zong et al., 2016). A NAT of MALAT1 was recently discovered and annotated as TALAM1. It contributes to the stability of MALAT1 by promoting the 3′ end cleavage and maturation of MALAT1. MALAT1 regulates the transcription and stability of TALAM1 through a feed-forward positive regulatory loop at the MALAT1 locus, that leads to high cellular levels of MALAT1 (Zong et al., 2016). On the other hand, NEAT1 is transcribed into two isoforms: a short form with a canonical poly (A)-tail (NEAT1_1) and a long unspliced form that lacks polyadenylation (NEAT1_2). NEAT1_2 is 22.7 kilobases (kb) and is processed by RNase P (Wilusz et al., 2012; Cornelis et al., 2016). Post cleavage by RNAseP, NEAT1_2 is processed into an RNA-stabilizing triple helix at its 3′ end and an unstable tRNA-like by-product (Statello et al., 2021).

## 4 Localization of LncRNA

Cellular localization of lncRNA can determine molecular contacts and hence its biological functions such as modulation of mRNA stability, signaling pathways, regulatory site of action and transcriptional expression of target gens. It can therefore be argued as a fundamental feature for understanding of lncRNA molecular mechanisms. Based on their localization, lncRNAs can be classified into the classes described below.

### 4.1 Nuclear lncRNAs

Nuclear localization of lncRNAs is governed by several factors such as splicing signals (Melé et al., 2017), polyadenylation signal at 3’ end (Rosenberg et al., 2015), nuclear retention elements and repeats (Zuckerman and Ulitsky, 2019; Guo et al., 2020). For example, in mouse embryonic stem cells (mESCs), splicing inhibitor peptidylprolyl isomerase E suppresses splicing of a subset of lncRNAs, leading to significant nuclear retention of many lncRNAs in mESCs (Guo et al., 2020). LncRNA CCAT1 (colon cancer-associated transcript 1) produces two isoforms: the long isoform (CCAT1-L) is nuclear and contains an internal polyadenylation site corresponding with the 3′ end of the short isoform (CCAT1-S), which is cytoplasmic (Xiang et al., 2014). In addition, lncRNAs can often contain nuclear retention signals or motifs that recruit nuclear factors, which promote the nuclear localization of the lncRNA. For example, the lncRNA maternally expressed gene 3 (MEG3) contains a 356-nucleotide nuclear retention element that associates with U1 snRNP (Azam et al., 2019). Repeat elements also drive lncRNA nuclear retention (Lubelsky and Ulitsky, 2018; Shukla et al., 2018). For instance, the lncRNA functional intergenic repeating RNA element (FIRRE) contains many unique repeats which promote FIRRE interaction with heterogeneous nuclear ribonucleoproteins (hnRNP) and chromatin localization (Hacisuleyman et al., 2016). In summary, nuclear lncRNAs regulate transcriptional expression of target genes in *cis* or in *trans* or at epigenetic level by being tethered to the chromatin in membrane-less nuclear domains.

### 4.2 Cytoplasmic lncRNAs

Cytoplasmic lncRNAs are expected to perform regulatory roles in the cytoplasm and may share the same processing and export pathways with mRNAs. Cytoplasmic lncRNAs are sorted into either organelles, or distributed in the cytoplasm, or interact with different RNA-binding proteins (RBPs) or microRNAs (Rackham et al., 2011). Linc-MD1 regulates muscle differentiation timing by sponging two miRNAs. LncRNA H19, Linc-MD1 and PTENP1 act as microRNA decoy of let-7, miR-133b and PTEN respectively to inhibit their transcription. LincRNA-p21 is both cytoplasmic and ribosomal lncRNA that negatively regulates JUNB and CTNNB1 translation in HeLa cells (Noh et al., 2018). LncRNA co-localization with ribosomes or endoplasmic reticulum (ER) regulates translation such as for ZFAS1 regulates mRNAs encoding proteins from the ribosomal complex. LncRNA UCA1 act as decoy of hnRNP1 to prevent it’s binding to p27-mRNA and restrict translation. Cytoplasmic lncRNAs like LINK-A, lnc-DC, NKILA and Lethe regulates different cellular signaling response. LINK-A activates BRK kinase and promote HIF1α stabilization. Lnc-DC promotes STAT3 phosphorylation, while NKILA inhibit NF-κB signaling and Lethe reduces RelA transcriptional turnover (Noh et al., 2018).

### 4.3 Mitochondria associated lncRNAs

Mitochondrial associated lncRNAs often function in regulation of mitochondrial metabolism, apoptosis and the crosstalk of mitochondria with nuclei (Zhao et al., 2018). For instance, survival associated mitochondrial melanoma specific oncogenic non-coding RNA (SAMMSON) is a lncRNA that maintains mitochondrial homeostasis, mitochondrial 16S *rRNA* maturation and expression of mitochondria-encoded polypeptides (Leucci et al., 2016). The mitochondria-encoded lncRNAs; lncND5, lncND6 and lncCytB form intermolecular duplexes with mRNAs and regulate their stability and expression (Statello et al., 2021). The RNA component of mitochondrial RNA-processing endoribonuclease (RMRP) is exported to the cytoplasm through its association with RBP HuR by exportin 1. RMRP is then exported to mitochondria, where it stabilized by G-rich RNA sequence-binding factor 1 (GRSF1), which allows its enrichment in the mitochondrial matrix (Quinn and Chang, 2016).

### 4.4 Exosome associated lncRNAs

Transcriptome analyses of human blood samples have identified many lncRNAs which localize into the exosomes through an unknown mechanism. Exosomal lncRNA like MALAT1 (Liu J. et al., 2021), growth arrest-specific 5 (GAS5) (Patel et al., 2022), H19 (Wang et al., 2020), HOTAIR (Yang et al., 2019), small ubiquitin-like modifier 1 SUMO1 pseudogene 3 (SUMO1P3) (Li et al., 2021b) and X-inactive specific transcript (XIST) (Lan et al., 2021) were previously shown upregulated in the blood, lung and breast tissues of cancer patients (Li et al., 2021b). It is noteworthy that the gut microbiota (Díaz-Garrido et al., 2021; Park et al., 2021), intracellular pathogens like *Salmonella* Typhimurium (Imamura et al., 2018), *Mycobacterium tuberculosis* (Liu M. et al., 2021) and obligate intracellular pathogens like *Chlamydia psittaci* (Radomski et al., 2019), *Rickettsia* spp. (Liu Y. et al., 2021) and *Anaplasma phagocytophilum* are internalized into host cells *via* endocytosis (Zhou et al., 2020). They communicate with DCs and phagocytes’ host-derived factors through the exosome for activation of antibacterial immunity. A transcriptome analysis of serum derived exosomes during *Orientia tsutsugamushi* infection showed downregulation of *Let7* family of microRNAs (a type of non-coding RNA). *Let7* regulates immune response and cytokine activation by modulating TLR-4 signaling pathway (Jiang et al., 2020). These findings suggest that exosomal lncRNAs may modulate host immune response to intracellular bacterial infections.

In summary, lncRNAs have complex spatio-temporal regulation within the cell that is linked to sequence encoded localization signals. All these findings may help in prediction of their mechanism of functioning in the cell.

## 5 Molecular mechanisms of functioning of lncRNA

As discussed previously, lncRNAs regulate different cellular functions at transcriptional, post-transcriptional, translational or epigenetic level depending on their sub-cellular localization (Statello et al., 2021). Briefly, they primarily function as:

### 5.1 Signals

**Figure 2:**
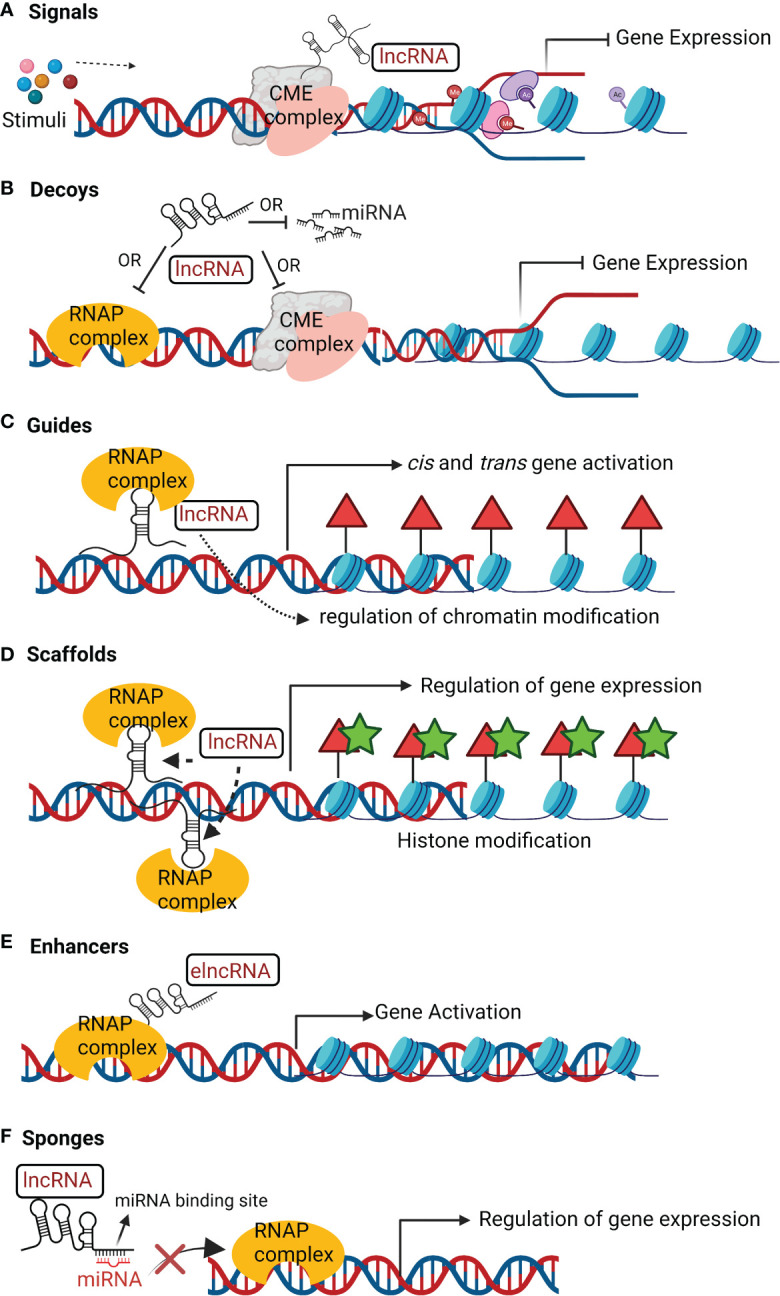
Mechanisms of action of lncRNAs. **(A)** Signals; lncRNAs receive a signal in response to stimuli to interact with chromatin-modifying enzyme (CME) complex to inhibit transcription. **(B)** Decoys; lncRNAs can interact with ribonucleoprotein (RNAP) complex or CME complex or miRNA to inhibit these regulatory factors from binding to the target gene and inhibiting its transcription. **(C)** Guides; lncRNAs can recruit RNAP complex and transcription factors to a specific genomic location and help to regulate chromatin arrangement for gene activation. **(D)** Scaffolds; lncRNAs can assemble several RNAP complex to inhibit or activate gene transcription. **(E)** Enhancers; enhancer lncRNA (elncRNA) enhance interaction of RNAP complex and chromatin to promote gene activation. **(F)** Sponges; LncRNA can act as sponges of miRNA. They have sequence complementary to miRNA and therefore can bind to miRNA, limiting its availability to transcription complex for regulation of target gene transcription.

LncRNAs which are expressed at a specific time and cellular location in response to stimuli are signal lncRNAs (**Figure 2A**). They can interact with CME such as histone methyltransferases to silence their target genes either by inhibition of transcription or through heterochromatin formation (Wang and Chang, 2011).

### 5.2 Decoys

Decoy lncRNAs act as transcriptional repressors in an indirect way by reducing the availability of the regulatory factors (**Figure 2B**) that includes: microRNA (miRNA), CME complexes, catalytic proteins, and TFs (Ahmad et al., 2021). For instance, the decoy lncRNA PANDA (P21 associated ncRNA DNA damage activated) regulates p53-dependent apoptosis. NFYA (Nuclear Transcription Factor Y Subunit Alpha) is a TF that activates transcription of apoptosis-related genes. PANDA binds to NFYA which prevents its binding to the promoter of apoptosis related genes (Liu J. et al., 2018). Taurine upregulated 1 (TUG1) and MEG3 are other instances of lncRNAs decoys miRNA from target mRNA (Balas and Johnson, 2018; Da et al., 2021). LncPRESS1 (lncRNA p53 regulated and ESC associated 1) is a pluripotency-associated lncRNA. lncPRESS act a decoy of deacetylase Sirtuin-6 and sequesters it from the chromatin, which promotes transcriptional activation by H3K56ac and H3K9ac of pluripotency-related genes (Jain et al., 2016).

### 5.3 Guides

LncRNA as guides (**Figure 2C**) regulate the expression of target genes by reprogramming CME complex or regulating the recruitment of epigenetic modifiers to the regulatory loci of target genes (Statello et al., 2021). For instance, KCNQ1 overlapping transcript 1 (KCNQ1OT1) and HOTAIR binds to PRC2, to regulate its target gene expression (Ahmad et al., 2021). LncRNA TCF7(transcription factor 7) recruits the SWItch/Sucrose Non-Fermentable (SWI/SNF) complex to the promoter of TCF7 that activates the Wnt signaling pathway to promote self-renewal of liver cancer stem cells (Gao et al., 2020).

### 5.4 Scaffolds

LncRNAs act as scaffold (**Figure 2D**) by providing structural support in assembly of regulatory protein complexes like ribo-nucleoprotein (RNAP) complex (Statello et al., 2021). They facilitate the assembly of RNAP complex to either transcriptionally inhibit or activate target protein-coding genes (Statello et al., 2021). For example, lncRNA TERC scaffolds the assembly of telomerase complex and associated accessory proteins with reverse transcriptase in a single RNAP (Wang and Chang, 2011). Under certain events, lncRNAs like MALAT1, ANRIL, and TUG1 act as molecular scaffolds to PRC1 and PRC2 to transcriptionally repress or activate target genes (Balas and Johnson, 2018).

Several lncRNAs act as scaffold for the assembly of nuclear condensates. Nuclear condensates are membrane-less compartments encompassing RNA-protein complex involved in regulation of cellular activities (Statello et al., 2021). NEAT1 act as scaffold for organization and function of paraspeckles (Jiang et al., 2017; Yamazaki et al., 2018). NEAT1_2 is essential for assembly of paraspeckles (Yamazaki et al., 2018). The middle region (8–16.6 kb) of NEAT1_2 comprises of two sub-domains of (~12–13 kb) and (~15.4–16.6 kb) length that act as scaffold to the paraspeckle core proteins NONO and SFPQ (Yamazaki et al., 2018). The mechanism behind the structural assembly of NEAT1_2 into the paraspeckles is not clear. LncRNA can also act as scaffold to bring distal chromatin regions into proximity. For example, FIRRE is transcribed from X chromosome. It functions in *trans* as scaffold with the nuclear matrix factor hnRNPU to maintain a nuclear domain (Lewandowski et al., 2019).

### 5.5 Enhancers

LncRNAs that act as enhancers can be broadly categorized into enhancer-RNAs (eRNAs) and enhancer-associated lncRNAs (elncRNAs). eRNAs are unstable, short, bidirectional capped and unspliced transcripts that lacks a 3’ poly-A tail (Statello et al., 2021). ElncRNAs are mostly unidirectional, 3’-polyadenylated and spliced. eRNAs can drive directly the expression of target genes through chromatin looping and interaction with scaffold proteins (**Figure 2E**). This interaction leads to regulatory contacts between enhancers and promoters of the distally located target genes (Statello et al., 2021). For instance, eRNA P53BER (p53-bound enhancer region) (Melo et al., 2013) and elncRNA SWINGN (SWI/SNF interacting GAS6 eRNA) (Grossi et al., 2020) recruit chromatin-activating complexes to the promoters of the target protein-coding genes. In response to oestrogen receptor (ER) transcription activation, eNRIP is another bi-directionally transcribed eRNA, that recruits cohesin to form chromatin loops. This interaction promotes contact between the eNRIP1 and the promoters of nuclear receptor interacting protein 1 (NRIP1) and trefoil factor 1 (TFF1) (Li et al., 2013).

### 5.6 Sponges

LncRNAs as sponges contain sites complementary to miRNA for interaction (**Figure 2F**). This results in reduced miRNA availability to target mRNAs (Statello et al., 2021). For instance, lncRNA PNUTS contains seven complementary sequences to miRNA-205, which reduces the availability of miRNA-205 to bind and inhibit mRNAs of the zinc finger E-box-binding homeobox 1 (*ZEB1*) and *ZEB2* (Grelet et al., 2017). The sponging of miRNA-205 by lncRNA PNUTS leads to increased expression of ZEB1 and ZEB2 which promotes epithelial to mesenchymal transition, and breast cancer cell migration and invasion (Grelet et al., 2017). LncRNA CCAT1 is sponge for miRNA-7 which upregulates the expression of the TF homeobox gene B1, thereby promoting the proliferation and metastasis of esophageal squamous cell carcinoma (Gao et al., 2020).

In conclusion, the review discusses different classes of lncRNA, their subcellular localization, and mechanism of action and how these characteristics determine the functionality of lncRNAs, their target genes expression and downstream immune signaling events in host cells.

## 6 Role of lncRNAs in regulation of immune response against microbial components

Macrophages and DCs are the central players of host innate immune defense against pathogens. They recognize specific pathogen-associated molecular patterns (PAMPs) on invading pathogens through their pattern recognition receptors (PRRs). PRRs like lipopolysaccharide (LPS), bacterial nucleic acids, lipoproteins and peptidoglycan upon host recognition trigger specific immune signaling pathways mediated either by TF, NF-κB or the Interferon regulatory factor (IRF) family of proteins (Li and Wu, 2021). IRF mediates selective expression of interferons (IFN) and AIM2 inflammasomes which leads to activation of apoptotic and cell autonomous immunity against intracellular pathogen (Li and Wu, 2021; Pant et al., 2022) like *Listeria monocytogenes*, *Legionella pneumophila* (Omotade and Roy, 2020), *Salmonella* Typhimurium, *Francisella* spp.*, Rickettsia* spp., *Anaplasma phagocytophilum* (Rikihisa, 2011) and *Orientia tsutsugamushi* (Deretic, 2011; Ko et al., 2013). Type-I IFN can also dampen inflammasome signaling and promote bacterial infection like *Mycobacterium tuberculosis* (Novikov et al., 2011). NF-κB activation usually results in acute activation of pro-inflammatory pathways. Infact, NF-κB mediated induction of host responses is known to be modulated by other pathogens like *Coxiella burnetii* to trigger anti-apoptotic pathways; dampen inflammatory cell damage; and promote selective survival in bacterial replicative niche (Lührmann and Roy, 2007).

Many aspects of signaling response are regulated both at transcriptional and post-transcriptional level which allows fine-tuning of immune response against infections (Yoshinaga and Takeuchi, 2019). Recent studies have shown lncRNAs involvement in regulation of differentiation and activation of macrophages, DCs and adaptive immune cells like T- and B- lymphocytes (Walther and Schulte, 2021). Several high-throughput sequencing studies have deciphered lncRNAs regulatory effect on immune response upon infection or PRR based stimulation *in vitro* and *in vivo* (Walther and Schulte, 2021). In addition, functional conservation of these immune signaling pathways in different infection models makes it essential to discuss the role of these lncRNAs in regulation of immune response, and further investigate their role in obligate intracellular bacterial infections.

### 6.1 LncRNAs in TLR- NF-κB dependent immunity

eLncRNA IL-1β and lncRNA IL-1β-RBT46 act as enhancers of IL1β after TLR4-MyD88-NFκB activation by LPS (Walther and Schulte, 2021). Nuclear-lncRNA PACER is a positive feedback regulator that acts as decoy of NF-κB p50 subunit to promote NF-κB p50/p65 heterodimer formation. This leads to immune gene expression in human macrophages in TLR-4 dependent manner (Krawczyk and Emerson, 2014). lncRNA CARLR is induced upon LPS-treatment which binds to p65 subunit of NF-κB to promote pro-inflammatory gene expression (Castellanos-Rubio et al., 2017). IL7-AS as described previously acts as chromatin modifier by association with p300 and SWI/SNF complex to drive expression of CCL2 and IL-6 in TLR-MAPK/NF-κB pathway (Walther and Schulte, 2021). LincRNA- tumor necrosis factor alpha-induced protein 3 (TNFAIP3) interacts with chromatin regulator HMGB1 to form HMGB1/NF-κB complex. This interaction facilitates NFκB binding to promoter region of IL-6 (Ma et al., 2017). LncRNA EPAV act as decoy of SFPQ protein from the p65 promoter of NF-κB to regulate NF-κB activation in mouse (Walther and Schulte, 2021).

**Figure 3:**
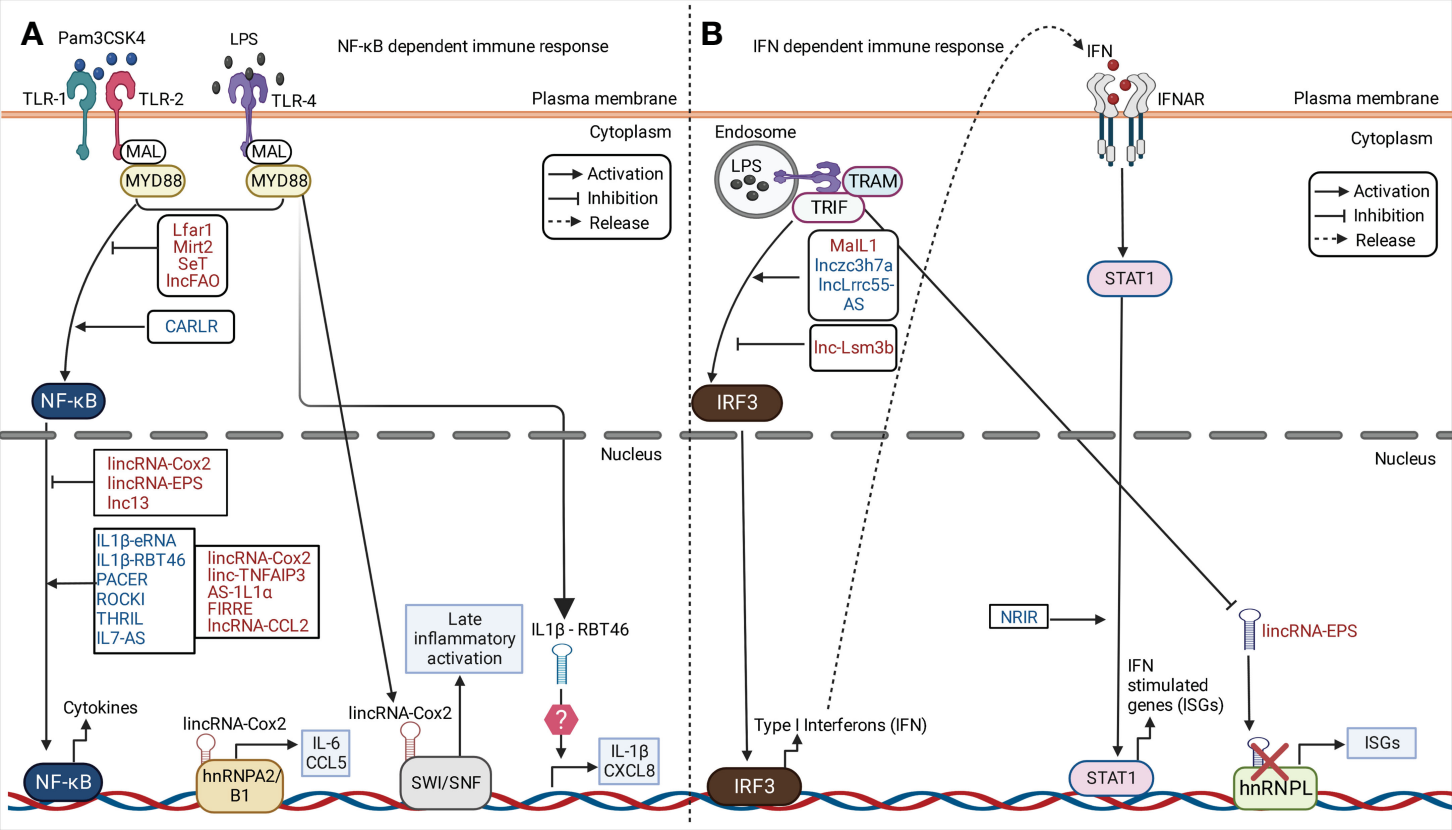
LncRNA in regulation of immune response against microbial components. Examples of cytoplasmic and nuclear lncRNAs acting as positive and negative regulators of NF-κB and IFN signaling pathway in PRR dependent pathway is illustrated in the figure. Examples of murine and human lncRNAs are represented in red and blue fonts respectively. **(A)** In response to Pam3CSK4 or LPS stimulation, lncRNAs positively or negatively regulate the activation of NF-κB signaling *via* TLR-1, 2 or 4. This leads to downstream regulation of cytokines expression. For instance, lincRNA-Cox2 is upregulated in murine phagocytes. It binds to heterogeneous nuclear ribonucleoprotein (hnRNP)A/B and hnRNPA2/B1 leading to both the activation and repression of different genes. LincRNA-Cox2 can bind the SWItch/Sucrose non-fermentable (SWI/SNF) complex, leading to activation of late inflammatory genes. Human IL-1β-RBT46 is upregulated following LPS stimulation, which leads to enhanced expression of IL-1β and CXCL8. **(B)** Upon LPS stimulation, lncRNAs like MaIL1, Lnczc3h7a, and lnc-Lsm3b positively and negatively regulate the activation of Interferon (IFN) regulatory factor 3 (IFN3) which leads to subsequent regulation of IFN production. lincRNA- EPS is downregulated following LPS stimulation leading to the upregulation of IFN stimulatory genes (ISGs).

LincRNA-Cox2 can act as positive and negative regulator of NF-κB through different mechanisms. It promotes IκBα degradation which causes translocation of NF-κB into the nucleus for pro-inflammatory cytokine expression (Xue et al., 2019). It negatively regulates TLR-induced ISG CCL5 expression by binding to hnRNPA/B and A2/B1 in the nucleus and inhibiting RNA polymerase II recruitment to the CCL5 promoter in Pam3CSK4-stimulated cells (**Figure 3A**). In another study, *in vitro* assays revealed a physical association between NF-kB subunits (RelA and p50) and the SWI/SNF complex in LPS-stimulated murine macrophages. siRNA knockdown of lincRNA-Cox2 showed decreased association between NF-kB subunits and the SWI/SNF complex (Hu et al., 2016). These findings indicated lincRNA-Cox2 can bind the SWI/SNF complex to modulate chromatin remodeling, and NF-kB dependent transcription of CCL5 (**Figure 3A**). LncRNA IL1α-AS upon TLR activation with LPS, Pam3CSK4 or poly (I:C) in cells recruits RNA polymerase II to induce expression of IL1α (Chan et al., 2015). LncRNA FIRRE is induced upon macrophage treatment with LPS, which promotes hnRNPU dependent transcriptional stabilization of IL-1β, IL-12β and VCAM1(Lu et al., 2017). TMC3-AS1 negatively regulates IL-10 by interacting with p65 in the nucleus, which prevents its binding to the NF-κB in the promoter region of IL-10 in macrophages and intestinal epithelial cell lines (Ye et al., 2020).

LncRNAs can also dampen the activation of PRR induced NF-κB gene expression. lincRNA-EPS and GAPLINC are downregulated *in vitro* upon TLR-MyD88-NFκB activation (Walther and Schulte, 2021). LncRNA THRIL was previously shown to be downregulated upon Pam3CSK4 stimulation (**Figure 3A**). This study suggested THRIL forms a transcriptional complex with hnRNPL at the promoter of TNF-α to drive its expression (Li et al., 2014). LncRNA Mirt2 is a negative feedback regulator of TLR-MyD88 pathway that interacts with TRAF6 and inhibits its auto-ubiquitination and oligomerization in the cytoplasm (Du et al., 2017). LncRNA lncFAO is another negative regulator which binds to the mitochondrial enzyme HADHB that promotes fatty acid oxidation and downregulates IL-1β to reduce inflammation (Nakayama et al., 2020).

### 6.2 LncRNAs in IFN dependent immunity

A study showed macrophage interferon-regulatory lncRNA 1 (MaIL1) positively regulates TRIF-TBK1-IRF3 induced type-I IFN expression in human macrophages after treatment with LPS. MaIL1 activates type-I IFN production (**Figure 3B**) through formation of complex with optineurin in a TLR4-MyD88-NF-κB dependent manner (Aznaourova et al., 2020). This results in ubiquitin-dependent aggregation of the optineurin signaling platform and phosphorylation of TF IRF3 by TBK1(Aznaourova et al., 2020). In clinical settings, MaIL1 expression correlated with IFNβ levels in broncho-alveolar lavage from pneumonia patients (Aznaourova et al., 2020). LncRNA NRIR is highly expressed in LPS treated human macrophages in TLR-4 dependent manner to promote ISG (CXCL10) expression through an unknown mechanism (Walther and Schulte, 2021). Murine macrophage systems have identified an array of lncRNA in IRF activation such as lncRNA Lnczc3h7a. It acts as a molecular scaffold to TRIM25 ubiquitin ligase and RIG-I (Lin et al., 2019). LncRNA LRRC55-AS upon STAT-dependent activation promotes type-I IFN production (**Figure 3B**) by supporting de-methylation and inactivation of PP2A (IRF3 inhibitor) by the PME1 protein (Zhou et al., 2019). LncRNA LSM3B negatively regulates RIG-I signaling through binding with RIG-I monomers to limit IFN production (Jiang et al., 2018). LincRNA-EPS interacts with chromatin repressor hnRNPL at target promoters to inhibit cytokine, chemokine and ISG expression (**Figure 3B**). Upon TLR-MyD88-NF-κB activation, lincRNA-EPS is repressed which leads to immune gene activation (Atianand et al., 2016).

### 6.3 LncRNAs in phagocyte differentiation and polarization

Myeloid specific human transcript HOTAIRM1 was previously reported as upregulated during granulocytic differentiation and regulated expression of the myeloid differentiation markers CD11b and CD18 (Walther and Schulte, 2021). Morrbid negatively regulates expression of the pro-apoptotic factor Bim in *cis*, by promoting PRC2 binding to the Bim promoter (Kotzin et al., 2016). Morrbid deletion *in vivo* showed reduced counts of eosinophil, neutrophil, and monocytes suggesting its role in life span of myeloid cells. lnc-DC is a lncRNA which interacts with TF STAT3 to promote STAT3 phosphorylation by preventing its de-phosphorylation by SHP1 (Walther and Schulte, 2021). A study showed NEAT1 is required for LPS-dependent maturation of DCs (Dai et al., 2021). Lnc-M2 was previously reported to be upregulated in differentiated macrophages (activated with M-CSF, Th2 cytokines IL-4 and IL-13) compared to M0 macrophages in a STAT3-dependent manner. It forms a complex with protein kinase A (PKA) to promote its phosphorylation and M2 polarization (Chen Y. et al., 2020). These findings suggest roles of lncRNAs beyond PRR dependent immunity to developmental role in host myeloid cell differentiation, polarization and repair.

Besides the role of lncRNAs in immune cell response and differentiation, they are also engaged in regulation of epithelial cells, endothelial cells and fibroblast’s PAMP induced inflammatory responses (Agliano et al., 2019). The lncRNAs involvement in PRR induced phagocytic and DCs immune signaling pathways may also contribute to regulatory functions in the other cell types and adaptive immunity. However, these questions are unexplored and have not been discussed in this review.

Collectively, this section discusses several examples of lncRNA playing a central role in the regulation of immune responses to PAMPs and bacterial nucleic acids. However, a better understanding of the role of these lncRNAs during acute pathogenic infections across different species may provide critical insights into designing innovative therapeutic strategies to target these infections.

## 7 Role of lncRNAs during infection by intracellular pathogens

LncRNAs are either host-derived or expressed by pathogens and their response may play a crucial role in modulating bacterial infections. The general outcome of intracellular pathogenic infection may depend upon how these lncRNAs modulate target genes to either promote or restrict the pathogens’ replication inside host (Agliano et al., 2019).

In this review, we discuss the functions of lncRNAs by categorizing them in response to infections caused by (a) facultative intracellular pathogens, (b) obligate intracellular pathogens, and (c) commensals. It highlights the functions of discovered lncRNAs in infection models like *M. tuberculosis*, *L. monocytogenes* and *S.* Typhimurium. These discovered lncRNAs could also be investigated for their role in regulation of host immune response against different obligate intracellular bacterial infections. A list of these lncRNA in regulation of immune response to several bacterial infections is summarized in **Table 1** of the review.

**Table 1:** LncRNAs and mammalian immune response during bacterial infection.

LncRNA	Cell type		Nature of regulation	Immune response regulated	Mechanism of action	Cellular localization	Organism		Reference
*Salmonella* Typhimurium									
IFNG-AS1/Tmevpg1/NeST	CD4^+^ Th1, CD8^+^ T, NK cells		Up	IFN-γ regulation	Promotes histone methylation at the IFN-γ locus *via* interaction with WDR5	Unknown	Human, Mouse		(Gomez et al., 2013)
NEAT1_2	HeLa		Up	facilitates paraspeckle formation and immune gene expression	Reduced NEXT exosome mediated nuclear RNA decay	Nuclear	Human		(Imamura et al., 2018)
*Mycobacterium tuberculosis*									
lncRNA-CD244	CD8^+^ T cells		Up	Regulates TNF-α and IFN-γ expression	Interacts with chromatin modification enzyme enhancer of zeste homolog 2 (EZH2), which catalyzes H3K27me3 at promoters of IFN-γ and TNF-α to inhibit their production	Unknown	Human		(Wang et al., 2015)
PCED1B-AS1	CD14^+^ monocytes from Mtb patients		Down	Enhance autophagy and reduce caspase-3 expression to inhibit apoptosis	Sponge of miRNA-155 in macrophages to relieve its effect on Forkhead Box O3 (FOXO3) and Ras Homolog MTORC1 binding (Rheb)	Unknown	Human		(Fathizadeh et al., 2020)
NEAT1_1; NEAT1_2 (both isoforms of NEAT1)	Th cells from Mtb patients; Macrophages		Up	Regulates IL-6 expression and promotes Mtb intracellular replication	Unknown	Nuclear	Human		(Agliano et al., 2019)
LncRNA XLOC_012582	B cells		Up	positively regulates SOCS3 which led to increased cytokine expression in TB patients	Unknown	Unknown	Human		(Fathizadeh et al., 2020)
*Mycobacterium bovis* BCG									
lincRNA-EPS	Monocytes; Macrophages		Down	Inhibition of apoptosis and enhanced autophagy	Enhances signaling of JNK/MAPK pathway and IFN-γ production	Nuclear	Mouse		(Ke et al., 2020)
MEG3	Macrophages		Down	Increased autophagy and bacterial clearance	Unknown	Unknown		Human	(Pawar et al., 2016)
lincRNA-Cox2	Macrophages		Up	Bacterial clearance in TLR4-MyD88 dependent manner	Regulates M1 polarization/ nitric oxide production; mediates of NF-κB/p65/p50 complex to the iNOS promoter; increased interaction with SWI/SNF complex	Cytoplasm/Nuclear		Mouse	(Carpenter et al., 2013)
*Mycobacterium smegmatis*									
MEG3	Macrophages		Up	Decrease TGF-β expression and inhibit selective autophagy	Increase mTOR-mediated phosphorylation of ribosomal protein S6 kinase beta-1(p70-S6K) at Thr389 and decrease accumulation of p62	Unknown		Human	(Agliano et al., 2019)
*Listeria monocytogenes*									
AS-IL1α	Macrophages		Up	IL-1α regulation	RNA polymerase II recruitment to the IL-1α promoter to increase its expression	Unknown		Mouse	(Chan et al., 2015)
lincRNA-EPS	Macrophages; Dendritic cells		Down	Activation of pro-inflammatory response to limit infection in TLR4-MyD88-NF-κB dependent manner	Regulates ISGs, TNF-α and IL-6 *via* direct interaction with chromatin repressor hnRNPL	Unknown		Mouse	(Agliano et al., 2020)
lincRNA-Cox2	Macrophages		Up	Activates NF- κB dependent pro-inflammatory gene expression for bacterial clearance	Increase interaction with SWI/SNF complex and hnRNA-A/B and A2/B1	Cytoplasm/Nuclear		Mouse	(Carpenter et al., 2013)
lncRNA-SROS1	Macrophages		Down	Regulates JAK-STAT and MAPK pathways for bacterial clearance	miRNA-1 degrades Sros1, relieves CAPRIN1 binding to Stat1,leading to STAT1 translation and enhanced IFN-γ signaling	Unknown		Mouse	(Li et al., 2021a)
*Legionella pneumophila*									
MaIL1	Macrophages		Up	Activates type-I IFN production in TLR4-MyD88-NF-κB dependent manner for bacterial clearance	Forms a complex with optineurin (OPTN) to trigger ubiquitin-dependent phosphorylation of IRF3 by TBK1	Cyto		Human	(Aznaourova et al., 2020)
Linc01215	Macrophages		Up	Activates type-I IFN production in TLR4-NF-κB dependent manner for bacterial clearance	Unknown	Unknown		Human	(Aznaourova et al., 2020)
*Pseudomonas aeruginosa*									
MEG9	Bronchial primary epithelial cells from cystic fibrosis patients		Down	Unknown	Unknown	Unknown		Human	(Balloy et al., 2017)
BLACAT1	Bronchial primary epithelial cells from cystic fibrosis patients		Down	Unknown	Unknown	Unknown		Human	(Balloy et al., 2017)
MEG3 isoform-4	Macrophages;epithelial cells; lung		Down	Decoy of miR-138	Infection downregulates MEG3 isoform-4 to release miR-138 that inhibits IL-1β and reduce inflammation	Unknown		Mouse	(Li et al., 2018)
*Brucella* spp.									
Gm28309	Macrophages		Down	Act as sponge of miR-3068-5p	Activates NF-κB and TGF-β signaling leading to assembly of NLRP3 inflammasome and IL-1β and IL-18 secretion.	Cytoplasm		Human	(Deng et al., 2020)
IFNG-AS1/Tmevpg1/NeST	PBMC		Up	IFN-γ regulation	Promotes histone methylation at the IFN-γ locus *via* interaction with WDR5	Unknown		Human	(Gheitasi et al., 2019)
*Rickettsia conorii*									
NONMMUT013718	Macrophages, endothelial cells		Up	Enhancer of Id2 (inhibitor of DNA binding 2)	Interacts with the promoter region	Unknown		Mouse	(Chowdhury et al., 2019)
NONMMUT024103	Macrophages, endothelial cells		Up	EnhancerApol10b (apolipoprotein 10b),	Interacts with the promoter region	Unknown		Mouse	(Chowdhury et al., 2019)
*Anaplasma phagocytophilum*									
lincRNA-TUCP	PBMC		Unknown	Unknown	Overlaps with the TNF/LT gene locus; Potentially regulates its expression	Unknown		Human	(Rennoll-Bankert et al., 2015)
*Clamydia trachomatis*									
ZFAS1	HeLa cells		Up	Inhibits host cell apoptosis *via* induction of MAPK/p38 pathway	Silences ZFAS1; leads to decrease in pORF5 mediated apoptosis and bacterial virulence	Unknown		Human	(Wen et al., 2020a)
MIAT	HeLa cells		Up	Inhibits mitochondria mediated host cell apoptosis and promotes bacterial replication	Increases expression of Bcl-2, and reduces caspase-3 expression	Unknown		Human	(Luo et al., 2022b)
ZEB1-AS1	HeLa cells		Up	Inhibits apoptosis and promotes bacterial replication	Acts as sponge for miRNA-1224-5p;Transcriptionally activates MAP4K4 to inhibit apoptosis	Unknown		Human	(Luo et al., 2022a)
lncRNA-IRF1	HeLa cells		Up	Inhibits apoptosis and promotes bacterial replication	Unknown	Unknown		Human	(Luo et al., 2022b)
FGD5-AS1	HeLa cells		Up	Promotes DNA replication; Inhibits apoptosis and promotes bacterial replication	Positively regulates the expression of Wnt/β-Catenin *via* unknown mechanism	Unknown		Human	(Wen et al., 2021)

### 7.1 LncRNAs during infection by facultative intracellular pathogens

#### 7.1.1 Mycobacterium tuberculosis

**Figure 4:**
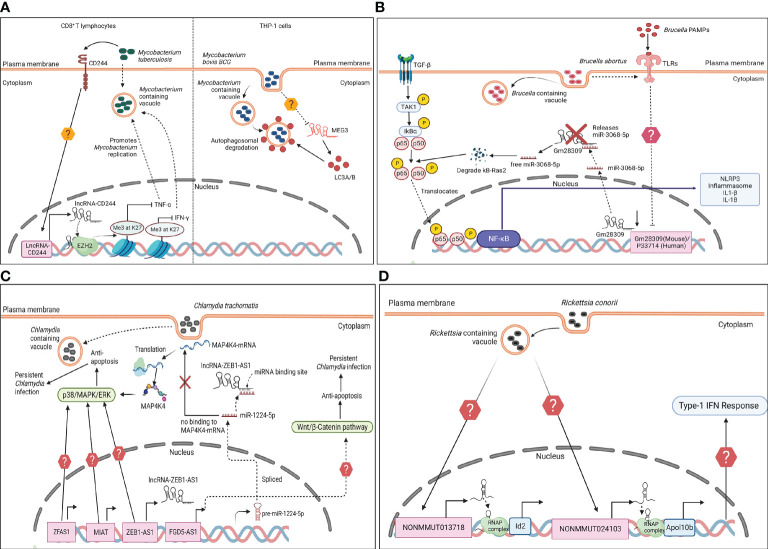
LncRNA in modulation of host immune response to facultative and obligate intracellular bacterial infection. **(A)** (left panel) During *Mycobacterium tuberculosis* infection, lncRNA-CD244 in human CD8^+^ T lymphocytes is upregulated in a CD244-dependent manner. LncRNA-CD244 interacts with the chromatin modification enzyme enhancer of zeste homolog 2 (EZH2), which leads to trimethylation of H3K27 at the tumor necrosis factor alpha (TNF-α) and interferon gamma (IFN-γ) loci. This represses the expression TNF-α and IFN-γ and promotes *Mycobacterium* replication. (right panel) Maternally expressed gene 3 (MEG3) is downregulated in THP-1 cells during *M. bovis* Bacillus Calmette–Guerin (BCG) infection. MEG3 increases LC3A/B conversion. LC3s decorate the *Mycobacterium* containing phagosomes and activate autophagy for bacterial clearance. **(B)** During *Brucella abortus* infection, PAMPSs on *B. abortus* activate the TLRs and TGF-β signaling. The TLRs *via* unknown mechanism inhibit expression of Gm28309 (LncRNA in mouse)/P33714 (human ortholog of Gm28309). Gm28309 sponges miR-3068-5p in steady state. During infection, downregulation of Gm28309 leads to release of miR-3068-5p in cytoplasm that degrades κB-Ras2 (an inhibitor of NF-κB) inducing p65 phosphorylation. In parallel, TGF-β activates TAK1 and IKK kinase mediates phosphorylation of IKB-α and p65, leading to translocation and activation of NF-κB signaling pathway. This leads to assembly of NLRP3 inflammasome, and secretion of IL-1β and IL-18. **(C)** *Chlamydia trachomatis* infection leads to upregulation of lncRNAs; MIAT, ZFAS1 and ZEB-AS1 *via* unknown mechanism. MIAT and ZFAS1 activate p38/MAPK/ERK signaling pathway through unclear mechanisms. ZEB1-AS1 activates p38/MAPK/ERK axis by acting as sponge of miR-1224-5p, which in turn cannot bind to MAP4K4-mRNA to inhibit its translation. FGD5-AS1 is also upregulated in response to infection. It activates the Wnt/β-Catenin pathway *via* unknown mechanism. The upregulation of these lncRNAs is linked to inhibition of apoptosis and increased *C. trachomatis* replication. **(D)** *Rickettsia conorii* infection in mouse phagocytic cells upregulates enhancer-lncRNA (elncRNA); NONMMUT013718 and NONMMUT024103 *via* unknown mechanism. They have been associated with an increase in the expression of Id2 (inhibitor of DNA binding 2) and Apol10b (apolipoprotein 10b). These genes contribute to activation of Type I-IFN signaling through unknown mechanisms.

Previous studies in TB patients and *in vitro* infected samples showed differential expression of several lncRNA and mRNA. Functional analysis of these transcripts showed majority of the lncRNA regulate immune signaling pathways such as TGF-β, IFN-γ, JAK-STAT; T- and B- cells differentiation and adaptive immune responses (Fathizadeh et al., 2020). PCED1B-AS1 is a lncRNA that act as sponge of miRNA-155 in macrophages to relieve its effect on Forkhead Box O3 (FOXO3) and Ras Homolog MTORC1 binding (Rheb). This results in inhibition of apoptosis and increase in autophagy to clear Mtb. Previously reported study on CD14^+^ monocytes isolated from TB patients showed downregulation of PCED1B-AS1 which led to enhanced autophagy and reduced caspase-3 expression to attenuate apoptosis (Yao et al., 2014). These findings proposed PCED1B-AS1 as an early diagnostic biomarker for Mtb infection (Fathizadeh et al., 2020). LincRNA-EPS was previously reported as downregulated in monocytes isolated from Mtb patients. Further a knockdown of lincRNA-EPS in RAW264.7 macrophages followed by infection with *M. bovis* (Mbv) BCG (Bacillus Calmette–Guérin) showed inhibition of apoptosis and increase in autophagy due to enhanced signaling of JNK/MAPK pathway and IFN-γ production (Ke et al., 2020). On the contrary, lincRNA-Cox2 silencing in RAW264.7 cells showed increased intracellular growth of Mbv BCG. LincRNA-Cox2 upregulation was linked to enhanced binding of NF-κB to the promoter of inducible Nitric Oxide Synthase (iNOS) and increases nitric oxide (NO) production and M1 macrophage polarization. This resulted in bactericidal activity against Mtb or Mbv infection (Fathizadeh et al., 2020). Previous studies have also shown pathogen-specific regulation by MEG3. MEG3 was downregulated in IFN-γ-treated THP1-derived-macrophages after infection with Mbv BCG, but it was upregulated in *M. smegmati*s (Msm) infected cells (Agliano et al., 2019). *In vitro* silencing of MEG3 showed increased conversion of LC3-I to LC3-II during Mbv BCG infection in human macrophages to induce autophagy (**Figure 4A**). The study also showed that MEG3 knockdown in cells resulted in reduced mTOR-mediated phosphorylation of ribosomal protein S6 kinase (p70-S6K) at Thr_389_ and increased accumulation of p62 compared to uninfected macrophages which is necessary for induction of selective autophagy (Pawar et al., 2016). In summary, the evidences suggest regulatory control by these lncRNAs in production of IFN-γ and autophagic clearance of Mtb or Mbv in the host.

Therapies to improve CD8^+^ T cell functioning is one of the promising strategy against Mtb infection (Fathizadeh et al., 2020). LncRNA-CD244 is found exclusively in humans and it overlaps with 5’-UTR of glutathione S-transferase theta-1 (GSTθ1) in CD8^+^ T cells (Wang et al., 2015). LncRNA-CD244 has been suggested as a potential target in anti-TB therapy. CD244^+^ CD8^+^ T cells isolated from peripheral blood mononuclear cells (PBMCs) of TB patients expressed higher levels of co-stimulatory molecule CD244 and lncRNA-CD244 compared to healthy controls through an unknown mechanism (Fathizadeh et al., 2020). Silencing of this lncRNA resulted in restricted Mtb growth. Moreover, immunoprecipitation studies in CD8^+^ T cells isolated from TB patients showed lncRNA-CD244 interacted with chromatin modification enzyme enhancer of zeste homolog 2 (EZH2) (**Figure 4A**), which catalyzed H3K27me3 at promoters of IFN-γ and TNF-α to inhibit their production (Wang et al., 2015). Previous studies have also shown that both the isoforms of NEAT1 were upregulated in T-helper (Th) cells derived from TB patients compared to healthy controls and its deletion resulted in reduced survival of Mtb in macrophages (Agliano et al., 2019). LncRNA XLOC_012582 positively regulates SOCS3 which led to increase in cytokine expression in B cells from TB patients (Fathizadeh et al., 2020).

RNA-sequencing studies from TB patients showed reduced expression of LncRNA-TGS1-1 and increased expression of miRNA-143 which represses downstream immune gene activation (Fathizadeh et al., 2020). Microarrays studies from plasma of TB patients have also identified NR_038221, ENST00000427151 and ENST00000354432 as potential biomarkers for early detection of TB (He et al., 2017).

#### 7.1.2 *Salmonella* Typhimurium

Nettoie Salmonella pas Theiler’s (NeST) or Tmevpg1 is an antisense lncRNA located within IFN-γ and is expressed in CD4^+^ Th1 cells, CD8^+^ T cells, and natural killer (NK) cells in humans and mice (Agliano et al., 2019). NeST expression is enhanced during STm infection and this promotes bacterial clearance *in vivo* (Gomez et al., 2013). Transgenic B10.S mice expressing either SJL/J^-^ or B10.S-derived NeST RNA demonstrated decreased STm pathogenesis compared to WT B10.S without NeST. This study further showed that co-transfection of NeST with WD repeat domain 5 (WDR5) cDNA in transgenic B10.S resulted in interaction of NeST with WDR5. These mice during STm infection showed increased H3K4me3 marks at IFN-γ locus in activated CD8^+^ T cells. Further, polymorphisms in NeST resulted in difference in T-cell mediated responses which modulated the inflammatory activities of cells to confer resistance against STm infection (Gomez et al., 2013). RNA-sequencing studies on STm-infected HeLa cells showed significant upregulation of NEAT1_2 compared to uninfected cells. NEAT1_2 was also induced in response to heat-killed STm which suggested that NEAT1_2 upregulation was as a result of PRR response of HeLa cells to STm PAMPs (Imamura et al., 2018). Knockout and knockdown of NEAT1_2 in HeLa cells resulted in reduced expression of TNF superfamily member 9 (TNFSF9) and CCL2 that led to increased susceptibility to STm infection. In normal HeLa cells, NEAT1_2 is readily degraded into unstable transcripts by MTR4 and RRP6 (components of nuclear exosome targeting (NEXT)-RNA exosome pathway). During STm infection, MTR4 and RRP6 levels is reduced leading to decreased degradation of NEAT1_2 (Imamura et al., 2018). Intracellular pathogens hijack metabolic pathways to foster replication in host cells. Recent research identified glucose transporters were encoded by genes SLC2A3 and SLC2A14. They are located near NEAT1_2 loci and were significantly upregulated in STm-infected HeLa cells and were essential for STm intracellular replication (Agliano et al., 2019).

#### 7.1.3 Listeria monocytogenes

Lm infection leads to increased expression of AS-IL1α which transcriptionally activates IL-1α by recruiting RNA polymerase II to IL-1α promoter (Chan et al., 2015). LincRNA-EPS was previously reported as downregulated during Lm infection. Further, lincRNA-EPS deficient BMDM and DCs when infected with Lm displayed reduced bacterial replication and increased expression of TNF-α, IL-6, and iNOS (Agliano et al., 2020). This concluded that lincRNA-EPS downregulation is essential for activation of NF- κB dependent pro-inflammatory response to limit Lm infection (Atianand et al., 2016). LincRNA-Cox2 is another well characterized lncRNA which is upregulated during Lm infection *in vitro* and *in vivo*. It also promotes Lm clearance *via* activation of NF- κB dependent pro-inflammatory gene expression through increased association with SWI/SNF complex and interactions with hnRNA-A/B and A2/B1 (Carpenter et al., 2013). Sros1 is a lncRNA that was reported as downregulated in IFN-γ-stimulated and Lm-infected macrophages. In uninfected cells, Sros1 regulates JAK-STAT and MAPK pathways (Li et al., 2021a). However, Lm infection activates miRNA-1 that degrades Sros1, which in turn relieves RNA binding protein Cell Cycle Associated Protein 1 (CAPRIN1) from Stat1. This stabilizes Stat1 transcription and increases STAT1 translation leading to enhanced IFN-γ signaling and bacterial clearance (Li et al., 2021a).

#### 7.1.4 Legionella pneumophila

Lp is an intracellular pathogen that employs a Dot/IcM Type IV secretion system (T4SS) apparatus to infect and replicate inside human phagocytes. Recent studies have discovered lncRNAs MaIL1 and Linc01215 are induced during Lp infection in a TLR4-NF-κB dependent manner to activate type-I IFN production and limit Lp replication inside macrophages. Concurrently, MaIL1 knockdown in macrophages resulted in an increased Lp intracellular replication. The growth could be restricted following treatment of Lp-infected macrophages with type-I IFN (Aznaourova et al., 2020). *C. burnetii* (Cb) is phylogenetically related to Lp and infects phagocytes in a similar mechanism using the T4SS. We hypothesize the signaling mechanisms involved in Lp pathogenesis would be similar in Cb infection. Although the type-I IFN response could counter or dampen Cb infection in a tissue-specific manner unlike Lp infection. It would be interesting to explore type-I IFN dependent lncRNA MaIL1 and other discovered lncRNAs in regulation of Cb pathogenesis.

#### 7.1.5 Pseudomonas aeruginosa

Transcriptome analysis of Pa-infected bronchial epithelial cells from patients with cystic fibrosis (CF) compared to non-CF epithelial cells revealed 108 differentially expressed lncRNAs. Of them, MEG9 and BLACAT1, and MEG3 were significantly downregulated (Balloy et al., 2017) MEG3 was also reported as downregulated in lung tissues from Pa-infected mice in TLR4-NF-κB dependent manner. Pa infection downregulates MEG3 isoform-4 which relieves miRNA-138. The miRNA-138 binds to IL-1β to reduce its expression, and thereby control inflammation and promote bacterial colonization *in vivo* (Li et al., 2018).

#### 7.1.6 Brucella abortus

Trancriptome analysis of THP-1 cells infected with *B. abortus* S2308 strain showed downregulation of lncRNA Gm28309 in a TLR4 dependent manner. The reduced expression of Gm28309 resulted in the activation of the NF-κB pathway and increased expression of TGF-β. TGF-β signaling activates TAK1 and IKK kinase mediated phosphorylation of p65. Also, Gm28309 act as sponge to miR-3068-5p in steady state. However, during infection miR-3068-5p is relesaed in the cytoplasm that binds and degrades κB-Ras2, promoting p65 phosphorylation (**Figure 4B**). The activation of the NF-κB pathway recruits the NLRP3 inflammasome, and secretion of IL-1β and IL-18 to modulate Ba infection *in vitro* (Deng et al., 2020). However, the exact role of Gm28309 in Ba pathogenesis is unclear. Another study reported increased expression of NeST and IFN-γ in PBMCs isolated from patients with brucellosis (Gheitasi et al., 2019).

### 7.2 LncRNAs during colonization by commensals

Commensals are obligate anaerobic opportunistic pathogens that constitute the human microbiota. They regulate many host cellular processes like metabolism, immune response, physiology and dysbiosis can lead to autoimmune disorders and microbiota associated cancers (Hou et al., 2022). LncRNAs are also emerging as a factor to regulate microbiota composition and host-microbiota interaction (Díaz-Garrido et al., 2021). Of them, A recent study has proposed lncRNA can act as molecular signatures in gut tissues and their spatio-temporal expression patterns could serve as biomarker to differentiate between a healthy and dysbiotic gut (Liang et al., 2015). It is noteworthy; these commensals triggered lncRNAs could also be explored in pathogenesis by obligate intracellular pathogens and therefore are discussed in this review.

One of the attributing factors to colorectal cancer (CRC) is overgrowth of *Fusobacterium nucleatum* (Fn). Recent trancriptome analysis of Fn-infected CRC cells revealed 43 upregulated lncRNAs that includes Keratin 7 antisense RNA (KRT7-AS) and endogenous retroviral-associated adenocarcinoma lncRNA (EVADR) (Chen S. et al., 2020). KRT7-AS promotes metastasis by CRC cells in nude mice by upregulating KRT7 in NF-κB dependent manner. CRC infection with Fn leads to upregulation of EVADR leading to increased metastasis of CRC cells both *in vivo* and *in vitro*. EVADR acts as scaffold to Y-box binding protein 1 (YBX1) leading to increased translation of SNAIL, SLUG, and ZEB1, which are required for epithelial to mesenchymal transition (EMT) (Lu et al., 2022). Another RNA sequencing analysis found 75 lncRNAs and 49 lncRNAs in probiotic and pathogen mediated carcinogenesis. Four lncRNAs: FRMD6-AS2, DIRC3, LIFR-AS1, and MRPL23-AS1 were proposed as unique signatures to CRC prognosis. LINC00355, KCNQ1OT1, LINC00491, and HOTAIR were associated with poor survival of CRC cells (Wen et al., 2020b). Fn is also an etiological agent for oral squamous cell carcinoma (OSCC). lncRNA MIR4435-2HG-5p was previously reported to be upregulated in Fn-infected OSCC cells. It act as sponge to miRNA-296-5p, leading to activation of serine/threonine kinase Akt2 and Akt2 induced tumorigenesis (Zhang et al., 2020).

Enterotoxigenic *Bacteroides fragilis* (ETBF) can induce tumor in dysbiotic gut. LncRNA *Bacteroides fragilis*-associated lncRNA1 (BFAL1) was previously reported to be upregulated in ETBF-infected CRC cells. BFAL1 over-expression promoted tumorigenesis by acting as sponge to microRNAs; miR-155-5p and miR-200a-3p, which in turn activated RHEB/mTOR pathway (Bao et al., 2019).

Lnc-GNAT1 was reported to be downregulated in *Helicobacter pylori* (Hp) induced gastric cancer. It negatively regulates the Wnt/β-Catenin signaling to regulate cancer cell proliferation (Liu L. et al., 2018). Lnc-SGK1 induces expression of glucocorticoid-inducible kinase 1 (SGK1) to induce Th2 and Th17 differentiation and inhibit Th1 differentiation in Hp-infected gastric cancer (Yao et al., 2016). Previous serological studies have also identified potential lncRNA signatures in Hp-infected cancer cells like H19, LINC00152, lncRNA AF147447, RP11-169F17.1, and RP11-669 N7.2 (Wen et al., 2020b).

### 7.3 LncRNAs during infection by obligate intracellular pathogens

Obligate intracellular pathogens include members of *Anaplasma*, *Rickettsia*, *Orientia*, *Wolbachia, Chlamydia*, and *C. burnetii.* Although lncRNA research in obligate intracellular pathogenesis is at nascent stage, recent studies have identified some lncRNAs role during infection and are discussed in the current section.

#### 7.3.1 Chlamydia trachomatis

Ct is an obligate intracellular pathogen which is a leading cause of sexually transmitted infection and reproductive complications. It infects host cells by modulating host cellular responses at transcriptional and translational level (Dzakah et al., 2021). Previous transcriptome analyses of Ct-infected HeLa cells have identified lncRNAs in inhibition of host cell apoptosis. For instance, lncRNA zinc finger antisense 1 (ZFAS1) was previously reported as upregulated in Ct infection. It was predicted to regulate MAPK pathways and its silencing led to decrease in *Chlamydia* plasmid protein pORF5-mediated-apoptosis and Ct virulence (**Figure 4C**). The study concluded pORF5 crucial role in activation of lncRNA ZFAS1, and ZFAS1 mediated induction of MAPK/p38 pathway to inhibit host cell apoptosis (Wen et al., 2020a).

A microarray analysis of IFN-γ-treated HeLa cells infected with Ct revealed 1718 lncRNAs as differentially expressed. Gene enrichment analysis showed these lncRNAs regulated host pathways involved in apoptosis, NOD-like receptor, TNF and RIG-I-like receptor signaling (Luo et al., 2022b). LncRNAs MIAT, ZEB1-AS1, and IRF1 were silenced in HeLa which resulted in increased apoptosis. MIAT-silenced cell lines showed reduced expression of Bcl-2, and increased activation of caspase-3. This indicated MIAT was involved in inhibition of mitochondria-mediated cellular apoptosis to promote Ct intracellular growth (Luo et al., 2022b). A follow-up study by Luo et al. studied the molecular mechanisms of ZEB-AS1 in Ct infection. siRNA mediated silencing of ZEB1-AS1 increased host cell apoptosis and impeded Ct growth. This was marked by downregulation of Bcl-2/Bax ratio that led to reduced mitochondrial membrane potential, release of cytochrome c, and caspase-3 activation. Further analysis revealed ZEB1-AS1 acted as a sponge for miRNA-1224-5p which transcriptionally activated MAP4K4 (**Figure 4C**). In conclusion, ZEB1-AS1 regulated miR-1224-5p/MAP4K4 axis to inhibit cellular apoptosis and promoted intracellular Ct infection (Luo et al., 2022a).

A previous microarray study also showed lncRNA FYVE, RhoGEF, and PH domain containing five antisense RNA1 (FGD5-AS1) was upregulated during Ct infection. Gene ontology analysis showed they enriched for DNA replication and Wnt signaling pathway. FGD5-AS1 positively regulated the expression of the Wnt/β-Catenin pathway (**Figure 4C**), inhibiting apoptosis and promoting intracellular replication of Ct in host (Wen et al., 2021).

#### 7.3.2 Rickettsia conorii

RNA-sequencing analysis to identify host lncRNA during *Rickettsia* infection was previously conducted by *Chowdhury et al.* C3H/HeN mice were infected with *R. conorii* (Rc) and transcriptome analysis was conducted from the infected lung tissues. This analysis identified 74,964 non-coding RNAs, out of which 206 lncRNAs were upregulated, and 277 were downregulated. The study identified two highly upregulated elncRNAs NONMMUT013718 and NONMMUT024103. NONMMUT013718 and NONMMUT024103 interacted with the promoter regions of Id2 (inhibitor of DNA binding 2) and Apol10b (apolipoprotein 10b) respectively to enhance their transcription (**Figure 4D**). Further, *in vivo* and *in vitro* infection with Rc showed increased expression of both lncRNAs and their associated genes in the macrophages. These findings were the first experimental evidence demonstrating regulatory role of lncRNAs in modulation of protective immunity against Rc infection (Chowdhury et al., 2019).

#### 7.3.3 Wolbachia

*Wolbachia* is an obligate intracellular pathogen that infects *Aedes aegypti*. Genome wide analysis of *A. aegypti* Aag2 cells infected with *Wolbachia* identified 3035 differentially expressed lncRNAs. Gene enrichment and network analysis revealed that the identified lncRNAs acted *in trans* to target genes and belongs to oxidative phosphorylation pathway. Further, competitive endogenous RNA (ceRNA) network analysis showed these lncRNA enriched for cellular oxidant detoxification pathway. Of them, lncRNA aae-lnc-7598 had the highest upregulation (Mao et al., 2022). Silencing of aae-lnc-7598 resulted in significant downregulation of antioxidant catalase 1B (CAT1B) gene and increase in mitochondria-mediated reactive oxygen species (ROS) in host cells. Another lncRNA, aae-lnc-0165 was downregulated after *Wolbachia* infection in host cells, which increased expression of REL1 through binding of aae-miRNA-980-5p, leading to Toll activation (Mao et al., 2022). Notably, these lncRNAs activate the anti-Dengue Toll pathway during *Wolbachia* infection. *Wolbachia* doesn’t infect human, but it can stay as an endosymbiont in vectors like *A. aegypti*. *A. aegypti* is a major vector responsible for transmission of *Dengue* virus. Taken together, we propose that these *Wolbachia* regulated lncRNAs could be explored as potential therapeutic markers to limit *Dengue* virus infection (Bensaoud et al., 2021; Mao et al., 2022).

#### 7.3.4 Anaplasma phagocytophilum

Ap infects the host *via* tick bite. It replicates within neutrophils and modulate host signaling responses mediated by secreted effectors such as AnkA. AnkA recruits HDAC1 to deacetylate H3 leading to transcriptional repression of several host immune response genes (Rennoll-Bankert et al., 2015). A previously reported AnkA-binding-CHIP sequencing analysis showed AnkA binding sites in the intergenic regions of target genes and transcriptionally regulated their expression *in cis.* NONCODE databases alignment showed AnkA binding sites in the promoter region of TNF/LT. The locus overlapped with a lncRNA named as lincRNA-TUCP. Further studies are required to dissect role of lincRNA-TUCP in regulation of TNF/LT locus during Ap infection (Dumler et al., 2016). There is also a limited understanding on how intracellular pathogens may regulate the expression of lncRNAs. AnkA effector of Ap was found to regulate the TNF/LT locus and the locus overlaps with linc-TUCP (Dumler et al., 2016). Future research could probe the prospective role of AnkA as a nucleo-modulin in regulating the linc-TUCP/TNF/LT locus during Ap infection. More studies should be conducted to investigate and identify bacterial effectors in regulation of host lncRNAs and their impact on downstream signaling pathways during infection.

## 8 LncRNAs as therapeutic targets

LncRNAs linked to host-pathogen interaction are promising targets for development of diagnostic and prognostic biomarkers. This possibility is supported by the clinical advantages represented by several of their molecular characteristics such as high tissue-specificity and regulation of specific biological targets in the host which circumvents undesired effects that arises from using protein-based drugs. Additionally, the non-translational nature, low endogenous expression and fast transcriptional turnover of lncRNA can result in greater biological effects with lower doses.

Some obligate intracellular pathogens and parasites infect *via* exosomes that are internalized by cells to form endosomes. Exosomes from gut microbes have been shown to improve metabolic functions (Li et al., 2021b). Conversely, fecal exosomes from mice infected with *Pseudomonas panacis* promotes glucose intolerance and insulin resistance (Li et al., 2021b). There is growing evidence on the regulation of cellular responses by exosomal lncRNAs. For instance, exosomal lncRNAs such as LNMAT2 (lymph node metastasis associated transcript 2), MALAT1, Gm26809 and NEAT1 etc. have been involved in metabolic reprogramming of tumor microenvironment (Sun et al., 2018), macrophage polarization and inflammatory diseases (Liu R. et al., 2018). Of note, MALAT1 over-expression suppresses macrophage activation, IL-10 response during *Leishmannia* and *Plasmodium* infection (Hewitson et al., 2020). Depletion of NEAT1_2 in HeLa cells promotes increased STm replication (Imamura et al., 2018). Furthermore, NEAT1_1 and NEAT1_2 knockout in macrophages have previously shown decreased IL-6 levels and delayed Mtb clearance (Huang et al., 2018). MALAT1 and NEAT1 are well characterized regulators of immune response in different pathogenic infection models. They may also be studied in regulation of inflammatory response, metabolic reprogramming and macrophage polarization during obligate bacterial infections. One advantage of targeting exosomal lncRNA is their stability inside exosomes, tissue-specificity and often their response is disease-specific. The criteria are ideal for development of lncRNA based biomarkers (Mosquera-Heredia et al., 2021). This may also lead to the development of highly specific disease markers leading to other research questions including- could these discovered lncRNAs be used to develop infection specific biomarkers for bioterror agents like *C. burnetii*?

The most recent technique of targeting lncRNA for therapeutics is based on the use of antisense oligonucleotides (ASOs). ASOs specifically binds lncRNA based on their sequence complementarities that induce RNase H mediated cleavage of lncRNA (Lai et al., 2020; Lee and Mendell, 2020). However, ASO mediated targeting of lncRNA in clinical settings hasn’t been successful due to *in vivo* toxicity and lack of proper delivery systems. Current researches have attempted to improve the pharmacological properties of ASOs by chemically modifying them to enhance the binding affinity to their target lncRNA, thereby increasing resistance to degradation by nucleases and reducing immunostimulatory activity (Seth et al., 2008). Fused aptamers may also be used for enhanced specificity in the intracellular delivery of oligo-based drugs (Dassie and Giangrande, 2013). Another strategy is the use of small molecules for targeting lncRNAs. This strategy of targeting lncRNA has been less successful because of the complexity associated with development of a high affinity small molecule that can bind lncRNA. Additionally, it requires identification of RNA motifs which can be only achieved from complete understanding of the lncRNA structure (Warner et al., 2018). Often, these lncRNAs fold into several molecular domains and can engage in different molecular interactions making it difficult for designing a small molecule targeting the lncRNA (Statello et al., 2021).

## 9 Conclusion

Advancements in RNA biology techniques, single cell sequencing and CHIP-seq assays have shown that lncRNAs are central regulators for innate immune activation, inflammasome assembly, and host-pathogen interactions. However, the field of study is challenging due to the complexities arising from the unstable nature of RNAs as well as lack of technological advancements in studying the 3-D conformation and the molecular interaction of RNA *in vitro* and *in vivo*. It is also becoming increasingly evident that their sequence, expression levels, processing, cellular localization, organization and interaction with other molecules can define their functionality. Another bottleneck in lncRNA research is the poor sequence conservation across species. Therefore, it becomes difficult to correlate and extrapolate results between human and mouse experimental systems. This is partially mitigated by initial screening, synteny and motif conservation analysis among the lncRNAs from different species. Additionally, the existing databases and computational tools require improvement in the current algorithms to not only precisely assess orthologs of lncRNAs but also predict their functional conservation and interactome across species (Zheng et al., 2021). One promising strategy to overcome this limitation could be the use of humanized mice models to study lncRNAs in response to infections. For instance, the role of lncRNA-CD244 in Mtb infection was studied in severe combined immuno-deficiency mice (SCID) mice engrafted with lncRNA-CD244-depressed human CD8^+^ T cells. Comparable studies can vastly expand the knowledge in the field.

The review of current literature suggests that lncRNAs regulate cell specification and disease. These functions require deeper understanding of physio-pathological processes so that they can be therapeutically targeted with high specificity. Cellular RNA may be a promising target for drug discovery. Synthesizing functionally relevant motifs of lncRNAs or the whole RNA complementary to a target host molecule for drug development may prove to be an effective strategy for lncRNA-directed therapeutics. For example, (a) lncRNAs as sponges of miRNAs or affecting the splicing mechanisms of a protein coding gene, or (b) compounds that could regulate biological processes in host for clinical development. The advantage of such lncRNA-directed therapeutics would be that these oligonucleotides may target complex and undruggable cellular targets. Overall, development of better tools for comprehensive molecular understanding of lncRNAs in regulation of immune responses and host-pathogen interaction might help in development of lncRNA-based therapeutics for intervention of infectious and inflammatory diseases.

## Author contributions

AA wrote the review. All authors contributed to the article and approved the submitted version.
